# Mucocutaneous adverse events to immune checkpoint inhibitors

**DOI:** 10.3389/falgy.2023.1147513

**Published:** 2023-03-02

**Authors:** Fiorinda Muhaj, Padmavathi V. Karri, Wylie Moody, Alexandria Brown, Anisha B. Patel

**Affiliations:** ^1^ Department of Dermatology, University of Texas MD Anderson Cancer Center, Houston, TX, United States; ^2^ Department of Dermatology, University of Texas Health Science Center- Houston, Houston, TX, United States; ^3^ Department of Internal Medicine, HCA Houston Healthcare West, Houston, TX, United States; ^4^ Department of Internal Medicine, Texas Health Presbyterian Hospital, Dallas, TX, United States

**Keywords:** dermatitis, immune checkpoint inhibitor, pruritus, drug rash, anti CTLA-4, anti PD-1, anti PD-L1, immunotherapy

## Abstract

Immune checkpoint inhibitors (ICIs) have revolutionized cancer therapy. Since the approval of ipilimumab in 2011, a total of nine ICIs have gained indications for various solid and hematologic malignancies. The expanding use of ICIs in oncology underscores the need for diagnosis and treatment expertise in immune related adverse events (irAE). Cutaneous toxicities are the earliest and most common irAE in this class of therapy. In addition to the more frequent reactions including vitiligo, lichenoid dermatitis, psoriasiform dermatitis, other less common skin toxicities including bullous dermatoses, neutrophilic dermatoses, and autoimmune dermato-rheumatologic diseases have been reported. Even though less than 3% of cutaneous irAEs (irCAEs) are classified as grade 3 or higher events, irCAEs can greatly impact quality of life. Appropriate management of irCAEs is critical to avoid unwarranted interruptions or discontinuation of lifesaving immunotherapy.

## Introduction

Immune checkpoint inhibitors (ICIs) are monoclonal antibodies that enhance T-lymphocyte response by targeting cytotoxic T-lymphocyte antigen 4 (CTLA-4), programmed cell death 1 (PD-1), and programmed cell death ligand 1 (PD-L1). Ipilimumab was the first ICI approved in 2011 for advanced melanoma. Since then, nine checkpoint inhibitors have approved indications for various solid and hematologic malignancies (Table 1) (1–3).

**Table 1:** Checkpoint inhibitors and approved indications (1–3).

Target	Checkpoint inhibitor	Indications
CTLA-4	Ipilimumab	CRC, HCC, melanoma, mesothelioma, RCC, NSCLC
	Tremelimumab	HCC
PD-1	Nivolumab	CRC, esophageal SCC, HCC, HL, HNSCC, melanoma, mesothelioma, NSCLC, RCC, urothelial carcinoma
	Pembrolizumab	Breast cancer, cervical cancer, CRC, CSCC, endometrial carcinoma, esophageal carcinoma, gastric carcinoma, HCC, HL, HNSCC, melanoma, mesothelioma, MCC, NSCLC, large B-cell lymphoma, RCC, SCLC, urothelial carcinoma
	Cemiplimab	BCC, CSCC, NSCLC
	Dostarlimab	Endometrial carcinoma
PD-1 plus LAG-3	Nivolumab plus relatlimab	Melanoma
PD-L1	Atezolizumab	Breast cancer, HCC, melanoma, NSCLC, SCLC, urothelial carcinoma
	Durvalumab	NSCLC, SCLC, urothelial carcinoma
	Avelumab	MCC, RCC, urothelial carcinoma

CTLA-4, cytotoxic T-lymphocyte antigen 4; CRC, colorectal cancer; PD-1, programmed cell death 1; HCC, hepatocellular carcinoma; NSCLC, non-small cell lung cancer; RCC, renal cell carcinoma; SCC, squamous cell carcinoma; HL, hodgkin's lymphoma; HNSCC, head and neck squamous cell carcinoma; CSCC, cutaneous squamous cell carcinoma; MCC, merkel cell carcinoma; SCLC, small cell lung cancer; BCC, basal cell carcinoma; PD-L1, programmed cell death receptor-1 ligand; LAG-3, lymphocyte activation gene 3.

Antibodies to the PD-1/PD-L1 or CTLA-4 axis lift the constitutional inhibitory immune response. This enhances anti-tumor lymphocyte activity, but also contributes to immune related adverse events (irAEs) in over a third of patients (4). While any organ system is susceptible, cutaneous irAEs (irCAEs) are among the first and most frequent to develop, affecting 30%–60% of patients (5–8). Appropriate diagnosis and management of irCAEs is associated with reduced use of immunosuppressive agents and continuation of lifesaving immunotherapy (8). As the approved clinical use of ICIs broadens, recognition and appropriate management of dermatological toxicities becomes increasingly important. In this article we provide an updated review of the clinical spectrum and management of irCAEs.

## Epidemiology

IrCAEs are not dose dependent and occur irrespective of underlying malignancy (6), although melanoma and renal cell carcinoma appear to portend a greater risk (4). Patient characteristics such as cytokine profiles and human leukocyte antigens may also predict irCAEs (7).

CTLA-4 inhibitors are traditionally associated with a higher incidence of irCAEs compared to PD-1/PD-L1 inhibitors (1, 5, 7, 9), 43%–45% vs. 18%–34%, respectively (5, 10). True incidence of irCAEs overall is difficult to ascertain, as outside of clinical trials, mild toxicities may be underreported. Additionally, the frequency of specific dermatoses may be confounded by nonspecific rash terminology used in ICI trials (11).

IrCAEs manifest earlier than other irAEs, usually within 3–6 weeks and 5–9 weeks after initiation of ipilimumab and PD-1/PD-L1 inhibitors, respectively (7). Most irCAEs are low-grade with less than 3% progressing to grade 3 or 4 reactions per Common Terminology Criteria for Adverse Events (CTCAE) v.5 grading (7).

Combination immunotherapy with CTLA-4 and PD-(L)1 inhibitors is increasingly utilized. This improves therapeutic efficacy but also increases the incidence of all and high-grade irAEs (1, 9, 12), including irCAEs (59%–72%) (7). Grade 3–4 irCAEs occur in 2%–3% of patients receiving monotherapy compared to 4%–10% of patients on combination regimens (6).

## Pathogenesis

Checkpoints maintain immune homeostasis and self-tolerance. PD-1 is expressed by T-cells, B-cells, natural killer cells, and tumor-infiltrating lymphocytes (2). Antigen-presenting cells and nonimmune cells, including tumor cells, express ligands PD-L1 and PD-L2 (3, 7, 9). The binding of PD-L1/PD-L2 to PD-1 prevents lymphocyte activation against self-antigens but it inadvertently enables tumor evasion (1, 2, 5). PD-(L)1 inhibitors disrupt this interaction, thereby promoting T-cell activity.

CTLA-4, which is expressed on activated T-cells, inhibits T-cell activation when bound to co-stimulatory molecule CD28 (1, 7). CTLA-4 inhibitors, ipilimumab and tremelimumab interfere with this inhibitory signal and allow for unopposed T-lymphocyte activation.

Investigative targets for immune inhibitor pathways include lymphocyte-activation gene-3 (LAG3), T-cell immunoglobulin and mucin domain-3 (TIM3), V-domain Ig suppressor of T-cell activation (VISTA), and B and T lymphocyte attenuator (BTLA) (9). Relatlimab, a LAG3 inhibitor, was recently approved in combination with nivolumab for advanced melanoma (3).

While enhancing anti-tumor activity, the pharmacological blockade of CTLA-4 and PD-1/PD-L1 promotes autoimmunity *via* activation of tissue-resident immune cells (5, 13). IrAEs can also arise from cross-reactivity between tumor cells and self-antigens on normal tissue (5, 13). There is evidence to suggest that photodamaged skin is more susceptible to irCAEs (5, 14). Ultraviolet-induced cellular injury and subsequent release of self-antigens creates a pro-inflammatory milieu where autoreactive T-cells are already primed before ICI exposure (5, 13). Though further studies are needed, oral nicotinamide may help delay the onset of irCAEs (14).

## Cutaneous adverse events

The most common irCAEs include pruritus, vitiligo, morbilliform drug, eczematous, lichenoid, and psoriasiform eruptions. Most irCAEs are mild and can be managed without discontinuation of immunotherapy (7, 15). Table 2 includes a summary of management of mucocutaneous adverse events in the setting of ICIs. Grade 1–2 eruptions can typically be managed with topical steroids, bland emollients, and oral antihistamines, whereas high-grade eruptions require more specific management based on the rash type (13, 16).

**Table 2:** Mucocutaneous irAEs and treatment recommendations.

IrCAE category	irCAE	Treatment	
Inflammatory	Morbilliform	Grade 1 • Medium to high potency topical steroids (triamcinolone 0.1% cream or ointment, betamethasone 0.05% cream or ointment, clobetasol 0.05% cream or ointment) (13, 16) Grade 2 • First line: high potency topical steroids • Second line: systemic steroids (prednisone 0.5–1 mg/kg/day); nbUVB (13, 16)	
			Grade 3 or refractory: systemic steroids, tocilizumab, omalizumab, dupilumab (15–19)
	Eczematous dermatitis		Grade 3 or refractory: systemic steroids, omalizumab, dupilumab, patch testing (8, 16)
	Lichenoid dermatitis		Grade 2: acitretin, hydroxychloroquine Grade 3 or refractory: apremilast, acitretin, hydroxychloroquine, TNFi, IL17 inhibitor, cyclosporine, MTX
	Psoriasiform dermatitis	Grade 2: apremilast, nbUVB, acitretin ^(^5, 8, 16) Grade 3 or refractory: MTX, anti IL17-, IL-23-, IL-12/23 inhibitor, TNFi (15, 16, 18)	
Vitiligo	First line: photoprotection Second line: nbUVB, topical steroids, topical JAK inhibitors		
Pruritus		Grade 1: medium to high potency topical steroids, menthol-camphor lotion, capsaicin lotion (16) Grade 2: oral antihistamines or GABA analogs (16) Grade 3 or refractory: doxepin, SSRIs, aprepitant, naloxone, oral steroids, dupilumab, omalizumab, nbUVB (8, 16, 19)	
Antibody mediated	Bullous pemphigoid	Grade 1	
	Lichen planus pemphigoides	• high potency topical steroids (16) Grade 2/3	
		• high dose systemic steroids, doxycycline +/- nicotinamide • omalizumab, rituximab, IVIG, dupilumab, methotrexate, mycophenolate mofetil (5, 10, 15, 18–20)	
SCARs	SJS/TEN	Discontinue ICI + supportive care Consider high dose systemic steroids, IVIG, TNFi, cyclosporine, plasmapheresis, tocilizumab, mycophenolate mofetil (1, 5, 6, 15, 18)	
	DRESS	Discontinue ICI + supportive care High dose systemic steroids	
Dermato-rheumatologic	Vasculitis	Cutaneous disease: high potency topical steroids, hydroxychloroquine Systemic disease: systemic steroids, hydroxychloroquine, MTX, rituximab	
	Dermatomyositis	Cutaneous disease: photoprotection, topical steroids, topical calcineurin inhibitors (10) Systemic involvement: systemic prednisone, hydroxychloroquine, IVIG, MTX, mycophenolate mofetil (10)	
	Lupus		
	Morphea	Localized: high potency topical steroids, intralesional steroids Generalized: systemic steroids, phototherapy, MTX, mycophenolate mofetil (21, 22)	
	Cutaneous sarcoid/granulomatous dermatitis	Grade 1: medium to high potency topical steroids (16) Grade 2: high potency topical steroids, intralesional steroids, hydroxychloroquine (16) Grade 3: hydroxychloroquine, TNFi, doxycycline (16)	
Oral	Lichenoid mucositis	First line: topical corticosteroids (fluocinonide 0.05% gel, dexamethasone 0.1 mg/ml solution) (16) Second line or refractory: holding ICI, systemic steroids; for lichenoid mucositis systemic management as in lichenoid dermatitis	
	Sicca syndrome	First line: dental care, saliva substitutes (16) Second line or refractory: systemic steroids (23, 24)	
Hair	Alopecia areata	First line: high potency topical steroids (8, 19) Second line: intralesional steroids, topical JAK inhibitor, topical minoxidil (8, 19)	
Nails	Psoriasiform or lichenoid nail changes	First line: topical or intralesional steroids (25) Second line: retinoids, systemic biologics	
	Plate thinning, onycholysis, onychorrhexis, splitting	Nail hygiene, careful clipping, avoiding cuticle trimming, nail strengthening lacquer (16)	
Follicular	Acneiform eruption, rosacea, hidradenitis suppurativa	Grade 1: topical steroids, topical antibiotics (clindamycin solution) Grade 2: systemic antibiotics (doxycycline, minocycline) Grade 3: systemic retinoids (19, 26–28)	
Neutrophilic dermatoses	PG	First line: high potency topical or intralesional steroids Second line or refractory: systemic steroids, dapsone, colchicine, TNFi (29)	
	Sweet syndrome	First line: systemic steroids (10, 19) Refractory: dapsone, colchicine (10, 19)	

irCAE, immune related cutaneous adverse event; ICI, immune checkpoint inhibitor; CTLA-4, cytotoxic T-lymphocyte antigen 4 (CTLA-4); PD-1, programmed cell death 1; PD-L1, programmed cell death ligand 1; nbUVB, narrow band ultraviolet B phototherapy; TNFi, TNF-alpha inhibitor; MTX, methotrexate; JAK inhibitor, janus kinase inhibitor; SCARs, severe cutaneous adverse drug reactions; GABA, gamma-aminobutyric acid; SSRIs, selective serotonin reuptake inhibitors; IVIG, intravenous immunoglobulin; PG, pyoderma gangrenosum.

### Morbilliform/maculopapular eruptions

Morbilliform eruptions are the most frequent irCAE seen in 49%–68% of patients on CTLA-4 inhibitors and 20% of patients on PD-1/PD-L1 inhibitors (1, 10, 17). Onset occurs in the first 3–6 weeks after ICI initiation (1, 7, 17). Clinical findings include variably pruritic coalescing macules and papules. Histopathological examination reveals interface changes and a perivascular/periadnexal lymphocytic infiltrate with or without eosinophils (7, 18). Morbilliform rashes are usually low-grade and self-limited within 2–3 months (1, 5, 15). For high grade rash with intractable pruritus, prednisone 0.5–1 mg/kg/day can be given and tapered once the eruption improves to grade 1 (16, 18, 19). Steroid-sparing alternatives (SSAs) including narrowband ultraviolet light B (nbUVB), tocilizumab (anti-IL6R monoclonal antibody), dupilumab (anti-IL4Ralpha antibody), or omalizumab (anti-IgE monoclonal antibody) (15, 18) can be considered for severe presentations and chronic management.

### Pruritus

Pruritus, with or without rash affects 25%–36% of patients on CTLA-4 immunotherapy and up to 47% of patients on combination ICI (5, 6). Less than 3% of patients develop refractory grade 3 pruritus (5, 8). Onset occurs in the first 3–10 weeks of therapy (8). Basic laboratory workup is recommended for recalcitrant cases and should include eosinophil counts, serum IgE, renal, and hepatic prolife (8, 16, 19). Additionally, nonbullous phase of bullous pemphigoid (BP) should be considered and ruled out with skin biopsy and serologies (19).

Considerations for recalcitrant pruritus include biologics such as dupilumab and omalizumab, aprepitant (neurokinin 1 receptor agonist), low dose naltrexone, and systemic steroids (1, 8, 16, 19).

### Lichenoid eruptions

Lichenoid dermatitis is well documented with PD-1/PD-L1 inhibitors and less reported with ipilimumab therapy (1, 7, 8, 10). Most lichenoid reactions are mild with only 2% being grade 3 or higher (6). Pruritus is common and clinical presentation is heterogeneous ranging from flat-topped purple papules of classic lichen planus to a more morbilliform appearing rash (7, 8). Other variants include inverse, erosive, and hypertrophic lichen planus (LP) (5, 8). Genitalia, oral mucosa, and nails can be affected. Bullous lichen planus pemphigoides (LPP), pityriasis lichenoides chronica, and lichen sclerosus have been reported (7). Histopathology shows lichenoid infiltrate at the dermo-epidermal junction, basal vaculoar degeneration, and Civatte bodies (7, 8). Compared to idiopathic (LP), spongiosis is a feature seen more in ICI-induced lichenoid eruptions (1, 15, 17). Additionally, the inflammatory infiltrate is more closely related to the immune cell composition seen in acute graft-vs.-host-disease than to idiopathic LP (30).

For high-grade rashes, methotrexate, tumor necrosis alpha (TNF-alpha) inhibitors, cyclosporine, IL17 inhibitors, and apremilast have been used (8, 10, 17, 19). Importantly, lichenoid reactions may persist after immunotherapy discontinuation (19).

### Psoriasiform eruptions

Psoriasis secondary to anti-PD-1/PD-L1 therapy has an incidence of 12% and it occurs *de novo* or as exacerbation of pre-existing disease (1, 5, 6, 8, 15). De novo psoriasis is seen within 5–12 weeks of ICI initiation (8). Exacerbation of pre-existing disease occurs earlier and can affect up to 80% of patients (1, 17, 19). Plaque psoriasis is the most common but other subtypes including palmoplantar, pustular, erythrodermic, and inverse have been reported (5, 6, 8, 18). Importantly, psoriasiform reactions can be associated with psoriatic arthritis and uveitis (5, 6, 18). Histopathology shows epidermal acanthosis, parakeratosis, hypogranulosis with variable spongiosis (7, 18).

Grade 3 or higher eruptions require holding ICIs and consideration for methotrexate or systemic biologics, including IL17, IL23, IL12/23 antibodies, or TNF-alpha inhibitors (15, 18). In patients with active malignancy, broad immunosuppressants should be avoided and more targeted regimens pursued. TNF- and IL12/23-inhibitors have a higher risk of infection than newer biologics (10). IL23 and IL17 antibodies selectively inhibit Th17 axis cytokines, are minimally immunosuppressive, and have a rapid onset of action (1). However, IL17 inhibitors may exacerbate colitis as an irAE from ICIs (17). Though targeted SSAs are preferred, the effect of biologics approved in primary psoriasis has not been widely evaluated in oncology patients on immunotherapy (10, 19).

### Eczematous reactions

Eczematous dermatitis affects up to 20% of patients on PD-1/PD-L1 inhibitors and up to 68% of patients on anti-CTLA-4 agents (8). It has an early onset within 6 weeks of therapy (8). Clinical findings include ill-defined coalescing erythematous patches and papules with secondary skin changes and flexural predominance (5). Main histopathological features include epidermal spongiosis and minimal lymphocyte exocytosis with perivascular, lymphocytic infiltrate with eosinophils (7, 8).

For recalcitrant grade 3 rash, advanced therapy with dupilumab or omalizumab can be pursued (8).

### Vitiligo

Vitiligo is mostly reported in patients with metastatic melanoma (1, 5, 7, 8). In skin of color patients, it can have a significant psychosocial impact, particularly in cosmetically sensitive areas. In contrast to intrinsic vitiligo, it affects sun exposed surfaces more commonly and is less likely to koebnerize (1, 5). Poliosis can be seen at the same time (8). Onset is delayed, usually months after ICI initiation (8). Strict photoprotection is recommended. Though treatment is not required, topical steroids, topical janus kinase inhibitors, and nbUVB can be considered (31) Vitiligo usually persists after ICI discontinuation (1, 8).

### Immunobullous reactions

Bullous pemphigoid (BP) is well-documented in the setting of PD-1/PD-L1 inhibitors, with an incidence of 1%–5% (8). Onset can be delayed to months, making it one of the longer latency irCAEs (6, 7, 18, 19). ICI-induced BP presents similarly to intrinsic disease with tense bullae overlying erythematous plaques, accompanied or preceded by intractable pruritus (5, 15). Nonbullous manifestations are not uncommon and include pruritic urticarial papules and plaques. In contrast to idiopathic BP, mucosal involvement is common, up to 40% (8, 18).

As in classic BP, histopathology demonstrates subepidermal clefting with eosinophils. DIF shows linear deposits of IgG and C3 at the epidermal site or roof of the split (7, 8, 18). Antibodies against BP180 and less frequently BP230 are often elevated (8, 18).

BP may lead to ICI discontinuation in up to 70% of patients and can often be more resistant to treatment (30). As even small body surface area (BSA) involvement can be significant, high potency topical steroids are often used in conjunction with systemic therapies. First line systemic agents include oral steroids and doxycycline with or without niacinamide. An SSA should be started at presentation for grade 2 or higher eruptions to avoid ICI interruption and reliance on systemic steroids (20). Omalizumab, dupilumab, rituximab, intravenous immunoglobulin (IVIG), methotrexate can be used for grade 2–3 disease (5, 10, 15, 18–20). Importantly, BP can persist after discontinuation of ICIs emphasizing the need for selecting SSAs (1, 8).

LPP has overlapping features of BP and lichen planus. It has been reported mainly in the setting of nivolumab and pembrolizumab (32–34). Clinical features are heterogeneous with papules, plaques, erosions, and bullae on trunk and extremities. Oral involvement is common with one case series showing 50% (33). Histopathologic features include subepidermal blister with lichenoid or vaculoar interface dermatitis. DIF demonstrates linear IgG and C3 along the basement membrane zone. Serologies are positive for BP180 antibodies, while antibodies to BP230 have not been reported thus far (33–37).

Other less frequently reported blistering diseases include pemphigus vulgaris, mucous membrane pemphigoid, linear IgA bullous dermatosis, and dermatitis herpetiformis (6, 8, 18).

### Severe cutaneous adverse reactions (SCARs)

True Stevens Johnson syndrome and toxic epidermal necrolysis (SJS/TEN) from CTLA-4 and PD-1/PD-L1 inhibitors are rare but they portend poor prognosis with mortality rates of 10% for SJS and 50% for TEN (6, 18). Clinical manifestations include fever, malaise, and diffuse erythema progressing to flaccid bullae with epidermal detachment. Mucosal involvement affects the conjunctivae, genitalia, oral cavity, the respiratory, and gastrointestinal tract (5, 18). Skin biopsy reveals full thickness epidermal necrosis with a sparse dermal infiltrate (7, 15, 18).

In addition to discontinuing the culprit medication, supportive care, including ophthalmologic, gynecologic and urologic evaluation is critical. Admission to an intensive care or burn unit is recommended for extensive involvement. High dose steroids (prednisone or methylprednisolone 1 mg/kg/day) are usually administered and tapered once re-epithelialization occurs (16, 18). Intravenous immunoglobulin (IVIG), cyclosporine, tocilizumab, TNF-inhibitors, plasmapheresis, mycophenolate mofetil can also be used to halt progression (1, 5, 6, 15, 18).

PD-1/PD-L1 inhibitors can be associated with SJS/TEN-like eruptions, which unlike true SJS/TEN, evolve over weeks from milder morbilliform rashes (10, 18, 19). Thus it is crucial for patients with morbilliform eruptions to be monitored for red-flag symptoms, such as targetoid lesions or bullae that could indicate progression to a SCAR. SJS/TEN-like eruptions can also occur *de novo* weeks to months after ICI initiation (18). These reactions are milder than true SJS/TEN with less eye involvement and less denuded skin. Careful ICI rechallenge can be considered for SJS/TEN-like eruptions, if patients lack an alternative anti-cancer therapy (18). For true SJS/TEN, however, rechallenge is contraindicated (13, 18).

Drug reaction with eosinophilia and systemic symptoms (DRESS) to ICIs is rare and it presents with fever, generalized morbilliform eruption, and concurrent systemic irAEs such as hepatitis, colitis, azotemia (18). Management includes discontinuation of ICIs and high dose steroids tapered slowly over 6–8 weeks. SSAs such as TNF-inhibitors, tocilizumab, or dupilumab may be considered (18).

Acute generalized exanthematous pustulosis (AGEP) to ICIs is extremely rare. Pembrolizumab, ipilimumab, nivolumab, and atezolizumab have been implicated (18). AGEP presents within 48 h, with edema, erythema, and sterile pustules. Resolution is rapid after culprit withdrawal. Topical and systemic steroids (0.5–1 mg/kg/day) can be used and re-challenge can be considered (18).

### Dermatologic-rheumatologic diseases

Underlying connective tissue disease is not a contraindication to ICIs, though 50% of patients experience an exacerbation (19). Only a minority (20%–30%), however, will experience severe enough flares to warrant ICI discontinuation (13, 19, 23, 38).

Dermatomyositis has been reported to CTLA-4 and PD-1 inhibitors, though given the paraneoplastic association, exact incidence is unclear (17, 19, 39).

ICI-induced systemic lupus and lupus-like eruptions, including subacute cutaneous, bullous, and chilblain lupus have been reported in patients receiving PD-1 inhibitors or combination ICI (19, 24, 40). Interestingly, ICI-induced lupus is not female predominant and lupus autoantibodies are absent (24).

Scleroderma, eosinophilic fasciitis, and morphea (including localized plaque and generalized) have been linked to PD-1/PD-L1 inhibitors (8, 21, 22, 40–42). Underlying systemic sclerosis portends a risk for disease exacerbation including worsening skin thickening and renal crisis (43).

Vasculitis irAEs are rare, <1%, and have been reported from anti-PD1 or combination immunotherapy (40). The predominant types include giant cell arteritis, aortitis, or central nervous system vasculitis (23, 40). Reported smaller vessels vasculitides include leukocytoklastic vasculitis, type III cryoglobulinemia, granulomatosis with polyangiits and eosinophilic granulomatosis with polyangiitis (EGPA) (19, 23, 44–46). Evaluation for systemic involvement is required and often systemic steroids are initiated (19). Severe involvement may require plasma exchange or rituximab (24). Mepolizumab (IL5 antibody) was used in a case of EGPA from nivolumab (46).

Granulomatous reactions, including sarcoidosis or sarcoid-like reactions have been associated with anti-CTLA-4 and anti-PD-1/PD-L1 therapy with onset anywhere from two weeks to two years (6, 8, 9). Cutaneous sarcoidosis is seen more frequently in melanoma patients on ipilimumab (9). Other organs including the eyes, lymph nodes, and lungs can be affected (9, 17, 19, 40). ICI-induced sarcoid appears to have a mild course and in many cases discontinuation of ICIs may not be indicated (47).

### Hair and nail toxicities

Alopecia areata or universalis, poliosis, changes in hair texture, and less commonly hair repigmentation have been reported, though hair toxicities to ICIs are less frequent than with conventional chemotherapy (8–10, 19, 48–50). Eosinophilic folliculitis after nivolumab leading to scarring alopecia was described in one report (51). Though ICI-induced alopecia is rare (1% for PD-1/PD-L1 inhibitors and 5% for CTLA-4 inhibitors), it can greatly impact quality of life (8, 49, 50, 52). Severe disease may warrant systemic immunomodulators such as JAK inhibitors, though a risk-benefit analysis must be performed to avoid interference with immunotherapy. Some cases may spontaneously resolve (49).

Lichen planus and psoriasis of the nail can present with or without cutaneous disease (16, 25). Non-specific nail toxicities include plate thinning, onycholysis, onychorrhexis, and splitting (8, 16). Diffuse onychodystrophy and paronychia were reported with nivolumab (53). Two cases of clinical onycholysis presenting histologically with lichenoid changes were described, also in the setting of nivolumab (54).

### Oral mucosal toxicities

Compared to cytotoxic chemotherapy, the prevalence of oral toxicity is lower, affecting up to 7% of patients on PD-1/PD-L1 inhibitors (8, 52, 55). Lichenoid reactions followed by xerostomia are the most common (11, 48). Clinical findings include reticulated white patches, erythema, erosions, or gingival desquamation (8, 56). A case of lichenoid granulomatous stomatitis to nivolumab was reported, clinically mimicking lichenoid mucositis (57). Extensive involvement or recalcitrant symptoms may necessitate holding of ICI and systemic management as per cutaneous lichenoid eruptions.

One case of immune-mediated glossitis was described in association with pembrolizumab, subsequently improving on oral prednisone (58).

Anti-PD-1/PD-L1 induced sicca syndrome with xerostomia and parotid enlargement can mimic Sjogren's. SSA/SSB-antibodies, however, are absent (23, 24).

### Neutrophilic dermatoses

Sweet syndrome has been reported with ipilimumab in patients with melanoma (7, 17, 19) and less commonly with PD-1/PD-L1 inhibitors (7, 17, 19). Pyoderma gangrenosum (PG) has been infrequently associated with ipilimumab in 2 cases and pembrolizumab in one (7, 17, 19, 29).

### Follicular reactions

Follicular eruptions are less frequent and later in onset than with targeted therapy, nonetheless papulopustular rosacea, acneiform rash, and hidradenitis have all been reported, mainly to anti-PD-1 agents (19, 26–28).

#### Grading criteria

Grading follows the CTCAE, which focuses on BSA involvement (59). The severity of irCAEs should not be merely based on BSA but rather on symptoms and specific dermatosis. For instance, a morbilliform eruption with >30% BSA can be managed conservatively without ICI discontinuation in contrast to SJS with <5% BSA (10). A modified grading criteria produced by the American Society of Clinical Oncology focuses on symptoms and quality of life and appears more applicable to irCAEs (60) (Supplementary Tables S1, 9 S2).

## Summary

In general, irCAEs indicate a positive anti-tumor response (11, 12, 61). This has been well documented with vitiligo, which is predictive of response, progression-free survival, and overall survival in patients with metastatic melanoma (1, 7, 9, 12, 13). Progression free survival was seen to be higher in patients who experienced flares of psoriasis (18). Though the data is limited, alopecia was also found to have a positive tumor response (49).

Most irCAEs can be managed without discontinuation of ICIs. For high-grade eruptions that necessitate high doses of systemic steroids or other immunosuppressant, choice of therapy must be carefully evaluated to avoid dampening anti-tumor response (13, 18). There is increasing support for prioritizing targeted, non-steroidal immunomodulators with less T-cell impact (13, 18). However, there is lack of data on the effect of SSA, such as biologics, on immunotherapy underscoring the need for further studies in this area of oncodermatology.

## Author contributions

Substantial contributions to the conception or design of the work; or the acquisition, analysis, or interpretation of data for the work: FM, ABP, PVK, AB, WM. Drafting the work or revising it critically for important intellectual content: FM, ABP. Provide approval for publication of the content: FM, ABP. Agree to be accountable for all aspects of the work in ensuring that questions related to the accuracy or integrity of any part of the work are appropriately investigated and resolved: ABP. All authors contributed to the article and approved the submitted version.

## Conflict of interest

The authors declare that the research was conducted in the absence of any commercial or financial relationships that could be construed as a potential conflict of interest.
