# Supplementary material for: Mucocutaneous adverse events to immune checkpoint inhibitors
Source: Front Allergy. 2023 Mar 2;4:1147513. doi: 10.3389/falgy.2023.1147513 (PMC10017442; doi:10.3389/falgy.2023.1147513)
Supplement: Supplementary file 1 [file Table1.docx]

**Supplementary table 1: CTCAE v.5 grading ^59^**

| **Grade** | **1** | **2** | **3** | **4** | **5** |
| --- | --- | --- | --- | --- | --- |
| Rash | Macular or papular eruption covering <10% BSA with or without symptoms (e.g., pruritus, burning, tightness) | Macular or papular eruption covering 10–30% BSA with or without symptoms (e.g., pruritus, burning, tightness) and limiting of instrumental ADL | Macules/ papules covering >30% BSA with or without associated symptoms and limiting of self-care ADL | Generalized exfoliative, ulcerative, or bullous dermatitis | Death |
| Pruritus | Mild or localized, relieved spontaneously or by local measures | Intense or widespread, relieved spontaneously or by systemic measures | Intense or widespread, and poorly controlled despite treatment |  |  |
| Vitiligo | Hypopigmentation or depigmentation covering <10% BSA, with no psychosocial impact | Hypopigmentation or depigmentation covering >10% BSA or with associated psychosocial impact |  |  |  |
| Alopecia | Hair loss of up to 50% of normal for that individual that is not obvious from a distance but only on close inspection; a different hairstyle may be required to cover the hair loss, but it does not require a wig or hairpiece to camouflage | Hair loss of >50% of normal for that individual that is readily apparent to others; a wig or hairpiece is necessary if the patient desires to completely camouflage the hair loss or if loss is associated with psychosocial impact |  |  |  |

**Supplementary table 2: CTCAE modified grading criteria ^59, 60^**

| **CTCAE Term** | **Grade 1** | **Grade 2** | **Grade 3** | **Grade 4** | **Grade 5** |
| --- | --- | --- | --- | --- | --- |
| Skin and subcutaneous tissue disorders - Other, specify | Asymptomatic or mild symptoms; clinical or diagnostic observations only; intervention not indicated | Moderate; minimal, local or noninvasive intervention indicated; limiting age- appropriate instrumental ADL | Severe or medically significant but not immediately life- threatening; hospitalization or prolongation of existing hospitalization indicated; limiting self care ADL | Life-threatening consequences; urgent intervention indicated | Death |

Abbreviations: CTCAE, Common Terminology Criteria for Adverse Events; ADL, activities of daily living; BSA, body surface area
